# Mutation-based, neoadjuvant treatment for advanced anaplastic thyroid carcinoma

**DOI:** 10.3389/fendo.2025.1619875

**Published:** 2025-08-25

**Authors:** Sabine Wächter, Detlef K. Bartsch, J. Riera Knorrenschild, Anika Pehl, Friederike Eilsberger, Andreas Pfestroff, Markus Luster, Katharina Holzer, Andreas Neubauer, Elisabeth Maurer

**Affiliations:** ^1^ Department of Visceral, Thoracic and Vascular Surgery, Philipps University Marburg, Marburg, Germany; ^2^ Department of Hematology, Oncology and Immunology, Philipps University Marburg, Marburg, Germany; ^3^ Department of Pathology, Philipps University Marburg, Marburg, Germany; ^4^ Department of Nuclear Medicine, Philipps University Marburg, Marburg, Germany

**Keywords:** targeted therapies, immune checkpoint inhibitors, kinase inhibitors, anaplastic thyroid cancer, neoadjuvant treatment

## Abstract

**Introduction:**

The prognosis of anaplastic thyroid carcinoma (ATC) remains poor. Mutation-based targeted therapies and immune checkpoint inhibitors (ICI) have gained increasing importance in the treatment of advanced tumor stages. This study aimed to investigate whether mutation-based neoadjuvant therapy can convert an initially unresectable tumor into a resectable state, optimizing local tumor control and prolonging overall survival.

**Methods:**

Patients with stages IVB and limited IVC *BRAFV600E*-negative ATC received immediate combination therapy consisting of the multikinase inhibitor (mKI) lenvatinib and the immune checkpoint inhibitor (ICI) pembrolizumab upon diagnosis. Patients with *BRAFV600E*-positive tumors were treated with a BRAF/MEK inhibitor regimen, consisting of dabrafenib and trametinib. This neoadjuvant therapy was administered for 4–6 weeks before re-staging. If FDG-PET/CT imaging demonstrated tumor regression, surgical resection of the primary tumor was performed. In cases of limited distant metastases, these were also surgically removed. In the adjuvant setting, mutation-based systemic therapy was continued.

**Results:**

Between December 2021 and December 2024, a total of 14 patients were screened. Ultimately, 12 patients, with a median age of 73 years (range: 54–85), were treated with neoadjuvant therapy. At diagnosis, six patients had UICC stage IVB and six stage IVC ATC. A BRAFV600E-mutation was detected in two patients. Following neoadjuvant therapy, eight patients showed tumor regression, whereas three exhibited an inadequate response, characterized by disease progression or a mixed response on FDG-PET/CT. In one patient, therapy was discontinued early due to severe local symptoms. During neoadjuvant treatment, two cases of tracheoesophageal or tracheocutaneous fistulas were observed. Surgical resection was performed in nine patients. An R0 resection was achieved in two, an R1 resection in six, and an R2 resection in one patient. The median follow-up period was eight months (range 1–36). Median progression-free survival (PFS) was three months (range 1–not reached), while median overall survival (OS) was nine months (range 1–not reached).

**Conclusion:**

Neoadjuvant therapy for advanced ATC appears to be a promising treatment approach for a subset of affected patients. While initial results are encouraging, further research is needed to establish its precise role within the multimodal management of this aggressive malignancy.

## Introduction

Anaplastic thyroid carcinoma (ATC) is a rare but highly aggressive malignancy, with an incidence of only 1–2 cases per million people per year. Despite accounting for just 1–2% of all thyroid cancers (TC), ATC represents a significant proportion of TC-related mortality (1). Due to its rapid and invasive growth, the majority of patients present at diagnosis with either locoregional lymph node metastases and infiltration of adjacent structures (UICC stage IVB) or distant metastases (UICC stage IVC) (2, 3). Particularly in these advanced tumor stages, the prognosis remains poor, with a median overall survival (OS) of only 3–5 months (3).

Over the past two decades, ATC treatment has relied on a heterogeneous combination of therapeutic modalities, including surgery, external beam radiotherapy (EBRT), and chemotherapy (CTX) (4). However, surgical intervention is only considered, if the tumor is deemed resectable. Studies have demonstrated that achieving an R0 or R1 resection is associated with improved survival, whereas R2 or multivisceral resections in advanced disease stages do not significantly impact OS (5). Consequently, multivisceral resections are generally not recommended for UICC stage IVB tumors with upper aerodigestive tract infiltration or for UICC stage IVC tumors with distant metastases (1). Additionally, systemic therapies such as CTX and EBRT—whether alone or in combination—have historically yielded only transient and incomplete tumor responses, failing to significantly improve patient outcomes (6, 7).

Given these challenges, mutation-based personalized therapies have gained increasing importance, particularly in advanced or initially unresectable ATC (8–11). While these targeted therapies have primarily been used in palliative settings as second-line treatment following surgery and EBRT ± CTX, emerging evidence suggests a potential role in neoadjuvant treatment strategies (11–15).

Recent studies have demonstrated that in patients with *BRAFV600E*-positive ATC, neoadjuvant therapy with dual BRAF/MEK inhibition using dabrafenib and trametinib can convert an initially unresectable tumor into a resectable state, thereby prolonging progression-free survival (PFS) and OS (14, 15). For patients with *BRAFV600E* wild-type ATC, a combination of the immune checkpoint inhibitor (ICI) pembrolizumab and the multikinase inhibitor (mKI) lenvatinib has been shown to reduce tumor burden, alleviate associated symptoms, and facilitate subsequent tumor resection and locoregional disease control (12, 13).

In this study, we aimed to evaluate whether mutation-based neoadjuvant therapy can transform an initially unresectable ATC into a resectable state, resulting in improved local tumor control, prolonged PFS and OS.

## Materials and methods

### Study population and patient characteristics

This is a retrospective, single-center study, conducted at the University Hospital of Marburg. A total of 14 patients were screened for inclusion in this study. Two patients were not enrolled: one was excluded due to unexpectedly detected bilateral subdural hematomas during initial staging, which excluded mKI therapy. The other patient was not included because neoadjuvant therapy was delayed pending insurance approval. This patient passed away three weeks after their first visit to our clinic, before insurance approval was granted and treatment could begin. Ultimately, 12 patients were enrolled, including two with *BRAFV600E*-mutated ATC and ten with *BRAFV600E*-wildtype ATC.

All patients presented with unresectable disease and were treated at our hospital between December 2021 and December 2024. The diagnosis was histopathologically confirmed in all patients by two experienced pathologists.

The data of three patients included in our study (patient 1, 2 and 5) have already been published in the paper by Maurer et al. (10). Consequently, these cases are not newly reported here but are included for completeness and descriptive analysis. Furthermore, due to the long follow-up period, extended follow-up data are available for these three patients, which will now be reported.

### Pathology assessment (histological diagnosis, molecular pathological analysis)

Tumor debulking or biopsy for histological confirmation was performed prior to the neoadjuvant therapy. ATC diagnosis was histopathologically confirmed by two independent experienced pathologists prior to the commencement of treatment in all patients. Diagnostic analysis was performed on formalin-fixed and paraffin-embedded tumor tissue. Confirmation of tumor type was done using HE- and immunohistochemical staining. In addition, PD-L1 expression was determined in all patients.

For rapid identification of *BRAFV600E* mutation the PCR-based Idylla™ BRAF mutation test (Biocartis NV, Mechelen, Belgium) was used on all ATC patients included in this study.

Evaluation of further genomic alterations (including RET-/NTRK-/ROS1-fusions, *TP53*- und *TERT*-mutations, *RAS*-mutations, ALK-translocations etc.) in the tumor was done using next generation sequencing (NGS).

### Treatment strategy

For the reliable diagnosis of ATC core needle biopsy (CNB) or surgical tumor sampling is essential, as fine-needle aspiration (FNA) often fails to provide sufficient material due to the tumor’s undifferentiated nature and frequent necrosis. Only CNB or excisional biopsy ensures an adequate tissue sample for comprehensive histopathological evaluation, including molecular pathology. More detailed information on (molecular) pathological examinations are provided above. Before initiating neoadjuvant treatment, all patients underwent staging, including whole-body fluorodeoxyglucose positron emission tomography/computed tomography ([18F]-FDG PET/CT), esophagoscopy, and tracheoscopy to assess intraluminal tumor growth. Following the completion of staging and molecular pathological analysis, the patients were presented at an interdisciplinary tumor board meeting to determine the most appropriate treatment strategy.

In the majority of patients, neoadjuvant therapy with lenvatinib was initiated immediately after reference pathological confirmation of ATC, with minimal delay between diagnosis and treatment start.

For patients with a detected *BRAFV600E* mutation, off-label treatment with dabrafenib and trametinib was initiated. In contrast, patients with *BRAFV600E*-wildtype ATC received a combination of pembrolizumab and lenvatinib. While lenvatinib has been approved by the European Medicines Agency (EMA) since 2015 for the treatment of radioiodine-refractory thyroid carcinoma in Germany, the use of dabrafenib, trametinib, and pembrolizumab requires individual approval from health insurance providers. To avoid treatment delays, lenvatinib (14–20 mg daily) was initiated as a bridging therapy while awaiting insurance authorization. Upon approval, treatment was either escalated to lenvatinib in combination with pembrolizumab (200 mg intravenously every three weeks) or switched to dabrafenib (150 mg twice daily) plus trametinib (4 mg daily), depending on the molecular profile. This individualized, mutation-based therapy was administered for 4–6 weeks before re-evaluation. Adverse events during neoadjuvant treatment were classified based on the Common Terminology Criteria for Adverse Events (CTCAE), version 5.0.

Restaging was performed using [18F]-FDG PET/CT to assess tumor response to neoadjuvant therapy. Surgery was considered only if substantial tumor regression was observed, allowing for the possibility of an R0 or R1 resection without the need for multivisceral procedures, such as resection of the trachea, esophagus, or sternotomy. The extent of resection was determined based on the local tumor findings. If tumor growth was confined to a single thyroid lobe, a hemithyroidectomy was performed. For more advanced cases, a thyroidectomy was required. In instances of extrathyroidal extension, the resection included infiltrated surrounding lymphatic and fatty tissue, affected muscles, and, if necessary, the internal jugular vein. Lateral lymph node dissection was only performed, if there was clinical evidence of lymph node involvement.

Preoperative laryngoscopy was conducted to evaluate the risk of recurrent laryngeal nerve palsy, if not already present.

In cases where resectable limited distant metastases (at most pulmonary metastases) were present, these were surgically removed 2–4 weeks after primary tumor resection.

Postoperative complications were assessed according to the Clavien-Dindo classification (16).

Approximately two weeks after surgery, systemic therapy with either lenvatinib/pembrolizumab or dabrafenib/trametinib was resumed.

### Follow-up examinations

[18F]-FDG PET/CT was performed every 3–6 months to monitor disease status. Treatment response to combination therapies was evaluated based on RECIST 1.1 criteria. The effectiveness of neoadjuvant and systemic treatments was assessed by progression-free survival (PFS) and overall survival (OS). PFS was defined as the time from treatment initiation to structural disease progression, while OS was defined as the time from treatment initiation to death.

### Ethical approval

The study was conducted with the approval of the Ethics Committee of the University Hospital of Marburg (No.25–96 RS).

### Statistical analysis

Kaplan-Meier curves were used for OS and PFS. The demographic and treatment characteristics of the study group was reported as mean with standard deviation for normally distributed continuous variables and as median with interquartile range (IQR) for those with non-normal distribution.

Data were collected using Excel (Microsoft Office, Microsoft Corporation, Redmont, WA, USA).

## Results

Ultimately, between December 2021 and December 2024, the present study evaluated six patients with unresectable ATC stage IVC and six patients with locally advanced ATC stage IVB. The median age of the evaluated patients was 73 years (range: 54–85 years).

Among the patients with stage IVC carcinomas, five patients had disseminated pulmonary metastases, while one patient had disseminated cerebral, osseous, and splenic metastases. The Eastern Cooperative Oncology Group (ECOG) performance status ranged from 1 to 3, with five patients classified as ECOG 1, five as ECOG 2, and two as ECOG 3.

The demographic characteristics of the evaluated patients and pretherapeutic findings, including recurrent laryngeal nerve paralysis, thyroid lobe involvement, and tumor infiltration of surrounding structures as assessed by FDG-PET/CT, tracheoscopy, and esophagoscopy, are summarized in **Table 1**.

**Table 1:** Demographics of the neoadjuvant treated patients with ATC.

Median age at treatment start (range), years	73 (54-85)
Sex, *n* (%)	
Men	7 (60)
Women	5 (40)
Performance status, *n* (%)	
ECOG 1	5
ECOG 2	5
ECOG 3	2
UICC stage	
Non resectable IVB	6
IVC	6
Location of metastases, *n* (%)	
Lung	5 (85%)
Bone/Abdominal (Spleen, hepatic, pancreatic), Brain	1 (15%)
Preoperative recurrent laryngeal nerve palsy, *n* (%)	
Unilateral	5 (42%)
Bilateral	1 (8%
Thyroid involvement, *n* (%)	
Unilateral	3 (25%)
Bilateral	9 (75%)
Preoperative imaging parameters, *n* (%)	
Tumor infiltration of the carotid artery	1 (8%)
Internal jugular vein occlusion (tumor infiltration or tumor thrombus)	5 (42%)
Tumor infiltration of the trachea	8 (67%)
Tumor infiltration of the esophagus	3 (25%)

ECOG, Eastern Cooperative Oncology Group performance status; UICC, Union for International Cancer Control; 8th edition TNM staging (2017).

### Neoadjuvant treatment strategies

Immediately following diagnosis, treatment with lenvatinib 20 mg once daily was initiated in nine out of 12 patients. Two patients, aged 82 and 85, started with 10 mg/day, while one patient aged 58 began with 24 mg/day. **Table 2** presents the clinical data of the patients who received neoadjuvant therapy. Seven of the 10 patients with *BRAFV600E*-negative ATC were also treated simultaneously with the ICI pembrolizumab (200 mg every three weeks). In two patients, however, neoadjuvant treatment with pembrolizumab was not administered due to lack of reimbursement from their health insurance providers (patient 1 and 3). One patient received lenvatinib for only five days before requiring emergency operation (further information see below) due to worsening dyspnea and stridor (patient 11).

**Table 2:** Clinical data of the patients who received neoadjuvant treatment.

Pat	UICC stage	Age (years)	Neo-adjuvant treatment	Surgical intervention	R-status	Adjuvant/palliative treatment	PFS (months)	OS (months)
1	IVC	80	Lenvatinib 20mg	1. TTX + LAD + debulking 2. Thoracotomy, wedge resection middle lobe	R1	- Lenvatinib/ Pembrolizumab - Debulking - EBRT	3	7
2	IVC	57	Lenvatinib 20mg + Pembrolizumab	TTX + LAD	R1	- Lenvatinib/ Pembrolizumab, - - Carboplatin/ Paclitaxel	3	36 (still alive)
3	IVB	85	Lenvatinib 10mg	//	//	- Lenvatinib/ Pembrolizumab - BSC	//	12
4	IVB	82	Lenvatinib 10mg + Pembrolizumab	HTX + debulking + LAD	R1	- Lenvatinib/Pembrolizumab	3	6
5	IVB	73	Lenvatinib 20mg Dabrafenib + Trame-tinib	TTX + LAD	R1	- Dabrafenib/ Trametinib - Lenvatinib/ Pembrolizumab - Carboplatin/ Paclitaxel - Debulking - Tracheotomy - EBRT	3	32 (still alive)
6	IVB	69	Lenvatinib 20mg	HTX + debulking + LAD + reconstruction of the tracheocutaneous fistula with suturing of the trachea and muscle flap	R1	- Dabrafenib/ Trametinib - Tracheotomy - Lenvatinib/ Pembrolizumab	1 ½	20 (still alive)
7	IVC	73	Lenvatinib 20mg + Pembrolizumab	HTX + LAD + debulking	R1	- Lenvatinib/ Pembrolizumab	3	9
8	IVB	54	Lenvatinib 20mg + Pembrolizumab, EBRT, Tracheotomy	hemiclam-shell operation with TTX + debulking + reconstruction of the tracheoesophageal fistula with suturing of the esophagus, muscle flap and tracheotomy	R0	//	5	13
9	IVC	76	Lenvatinib 20mg + Pembrolizumab	//	//	- Lenvatinib/ Pembrolizumab - BSC	//	4
10	IVC	79	Lenvatinib 20mg + Pembrolizumab	//	//	- Lenvatinib/ Pembrolizumab - EBRT	8	9 (still alive)
11	IVC	63	Lenvatinib 20mg (5 days)	1. HTX left + debulking + LAD 2. HTX right + debulking + tracheotomy + thrombectomy of the superior vena cava	R2	- Lenvatinib/ Pembrolizumab	//	1 1/2
12	IVB	58	Lenvatinib 24mg + Pembrolizumab	TTX + debulking + LAD	R0	- Lenvatinib/ Pembrolizumab	No progress	4, still alive

Pat., Patient; OS, Overall survival; PFS, progression free survival; HTX, Hemithyroidectomy; LAD, Lymphadenectomy; Tumor debulking, resection of the strap muscles/sternocleidomastoid muscle/tumor-infiltrated jugular vein/soft tissue surrounding the thyroid gland; TTX, thyroidectomy; EBRT, external beam radiation therapy; BSC, best supportive care.

As the health insurance company rejected reimbursement, one of the two patients with *BRAFV600E*-positive ATC was treated exclusively with lenvatinib in the neoadjuvant setting (patient 6). In the other *BRAFV600E*-positive patient, neoadjuvant therapy with lenvatinib was switched to dabrafenib and trametinib once the molecular pathology results were available (patient 5).

**Figure 1:**
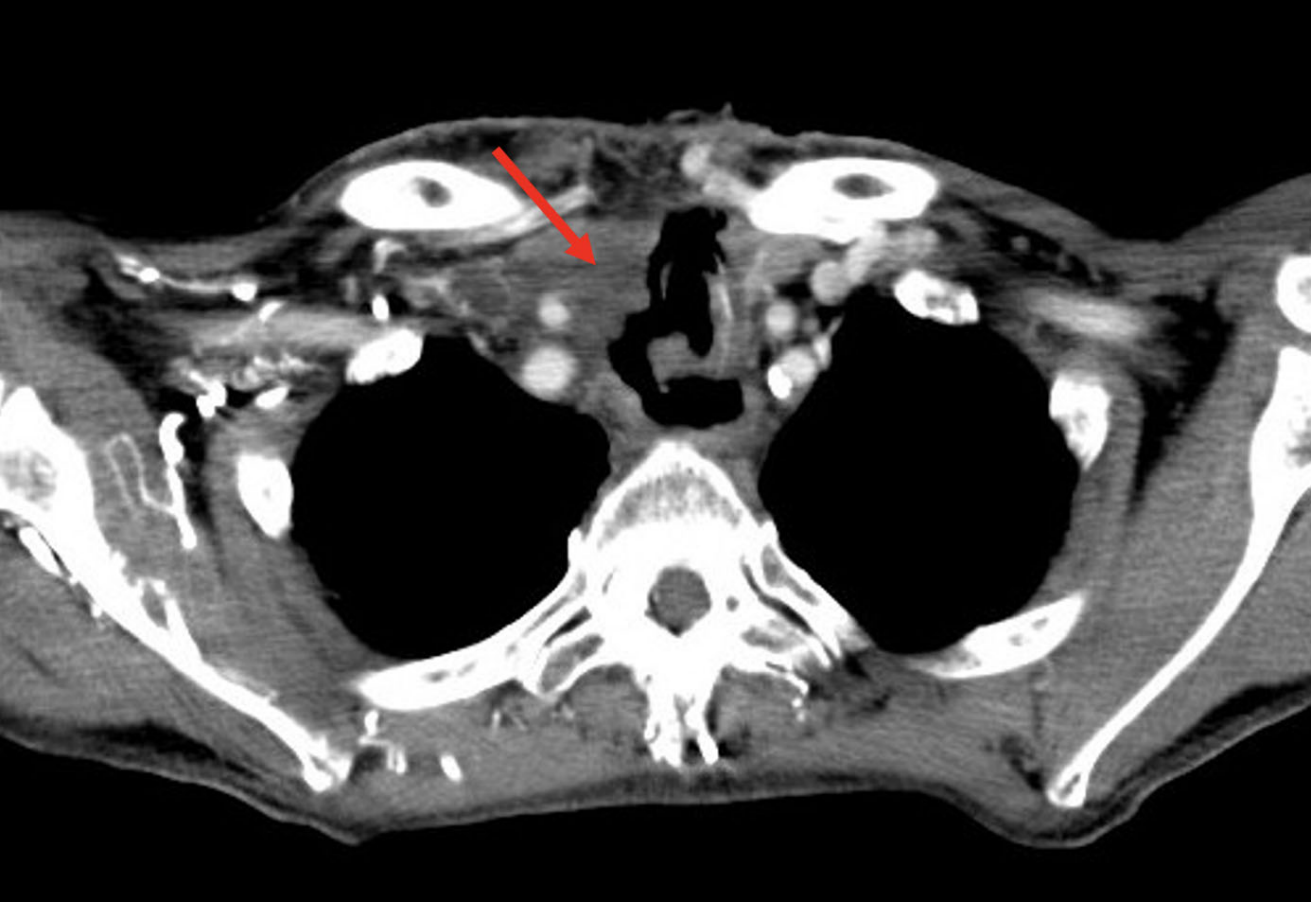
Esophagotracheal-cutaneous fistula following neoadjuvant therapy with lenvatinib and pembrolizumab, concurrent external beam radiotherapy (66 Gy), and tracheotomy (patient 8).

In one case, simultaneous EBRT of the neck region (66 Gy) was conducted in conjunction with neoadjuvant therapy with pembrolizumab and lenvatinib at an external hospital. This patient also underwent tracheotomy and percutaneous endoscopic gastrostomy (PEG) placement prior to starting the neoadjuvant therapy. Four weeks into this multimodal treatment, the patient developed esophagotracheal-cutaneous fistulas, leading to recurrent aspiration (patient 8, **Figure 1**).

### Neoadjuvant treatment efficacy

The median time from the initiation of neoadjuvant therapy to surgery was 28 days (range: 5–56). Due to non-compliance, one patient received neoadjuvant therapy with lenvatinib for 56 days (patient 6).

**Figure 2:**
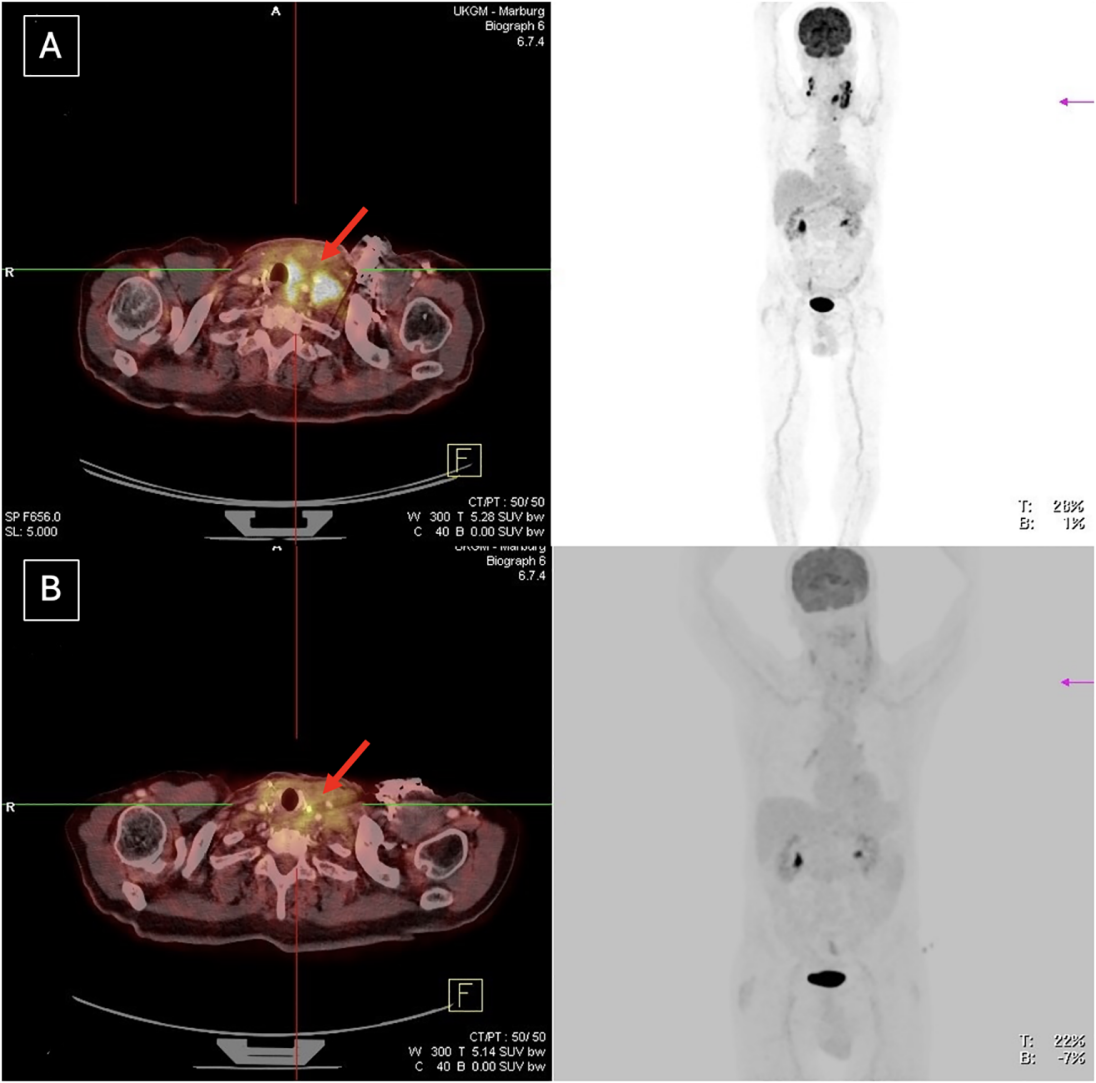
Adequate tumor response to neoadjuvant treatment in patient 5, with an R1 resection achieved. **(A)** Before neoadjuvant treatment **(B)** After neoadjuvant treatment.

**Figure 3:**
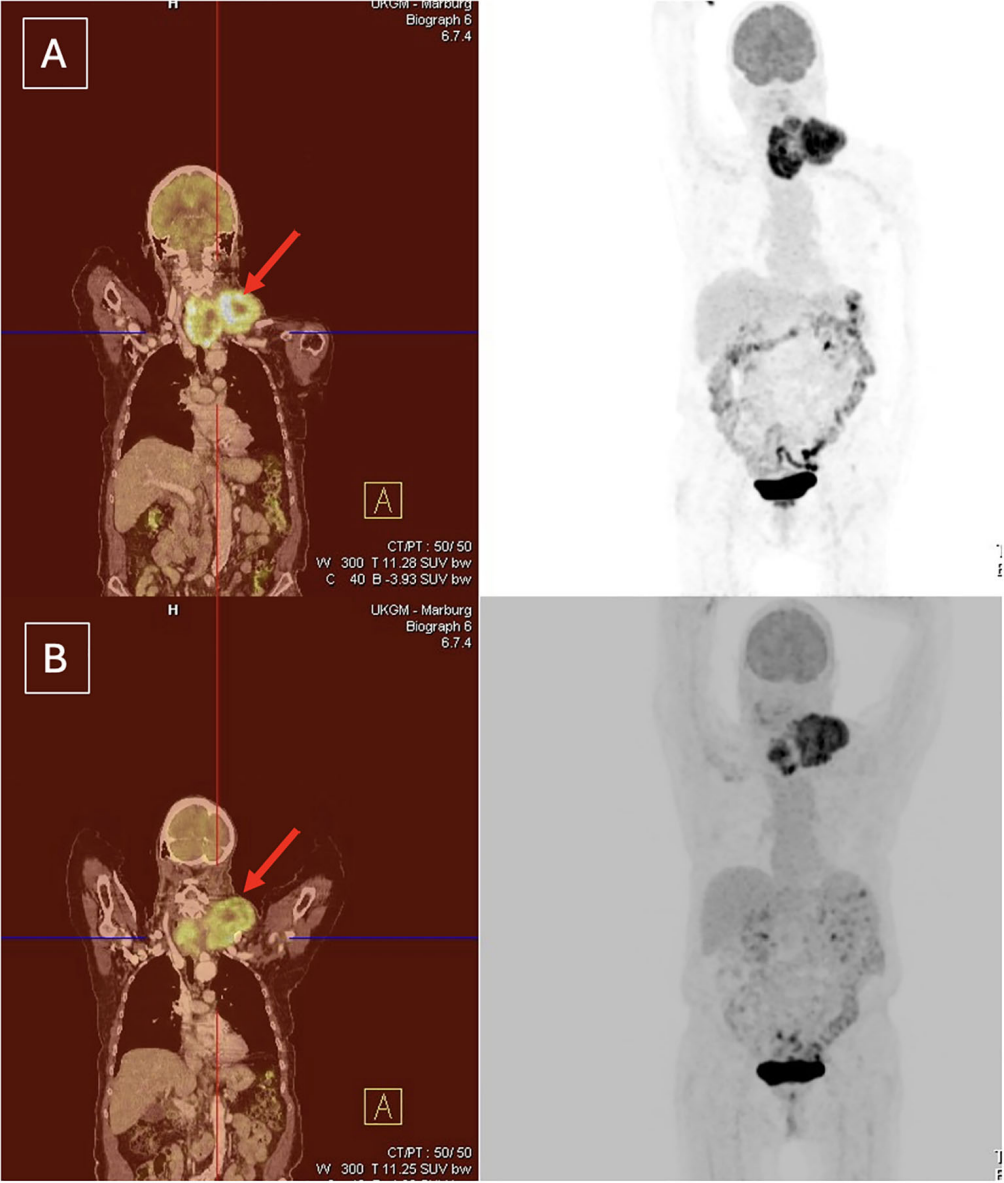
Insufficient imaging response to neoadjuvant treatment. Despite this, the patient underwent tumor debulking due to brachial plexus infiltration and worsening local symptoms (patient 7). **(A)** Before neoadjuvant treatment **(B)** After neoadjuvant treatment.

Pretreatment and preoperative imaging were compared to assess tumor size reduction and decrease in metabolic activity following neoadjuvant therapy. An example of an adequate response to neoadjuvant therapy in [18F]-FDG PET/CT is shown in **Figure 2**. Three patients showed an inadequate response, exhibiting progression or a mixed response (primary tumor vs. metastasis) on [18F]-FDG PET/CT (patient 3, 7 and 9). The poor response in one of these patients may have been related to reduced monotherapy with 10 mg lenvatinib/day due to age considerations (patient 3). As a result, two of these patients did not undergo surgery and continued systemic therapy with palliative intent. In one patient, despite insufficient imaging response, cervical exploration with tumor debulking was performed due to tumor infiltration of the brachial plexus and associated worsening local symptoms (patient 7, **Figure 3**). This patient underwent an R1 resection.

After neoadjuvant therapy, the extent of surgery varied. Three patients underwent hemithyroidectomy with lymphadenectomy and tumor debulking, which included resection of the strap muscles, resection of the sternocleidomastoid muscle and/or resection of the tumor-infiltrated jugular vein. Four patients had a thyroidectomy with tumor debulking. Due to favorable response of the pulmonary metastases to neoadjuvant therapy, one of these patients also required a right-side thoracotomy and wedge resection of the middle lobe to remove limited pulmonary metastases two weeks after cervical surgery (patient 1).

**Figure 4:**
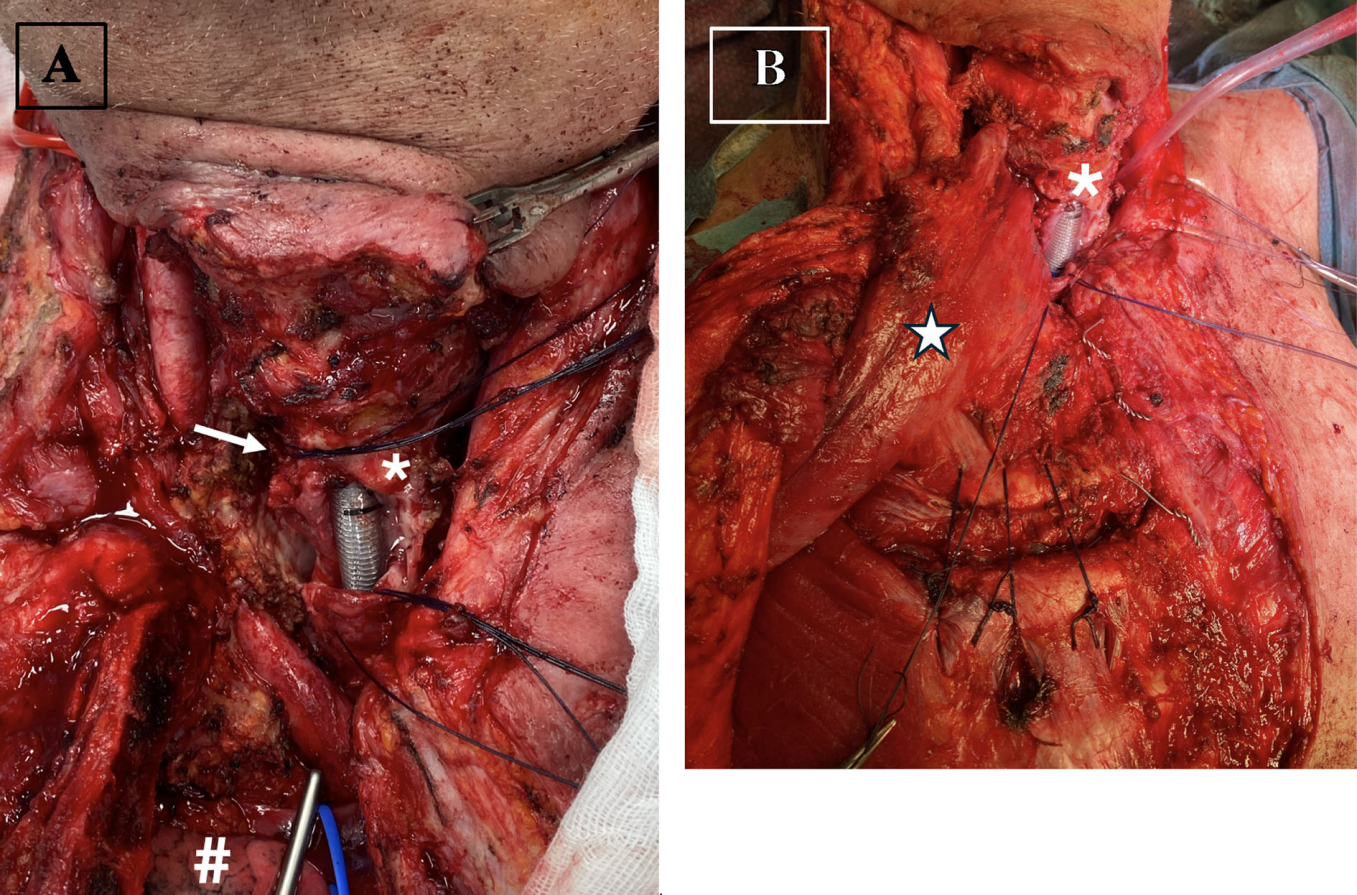
Intraoperative view of patient 8 with an esophagotracheal-cutaneous fistula following neoadjuvant therapy. The patient underwent a complex hemi-clamshell operation, including thyroidectomy, tumor debulking, reconstruction of the tracheoesophageal fistula with esophageal suturing, muscle flap placement, and tracheotomy. **(A)** Depiction of the esophagotracheal fistula prior to reconstruction **(B)** Coverage of the esophagotracheal fistula using a pectoralis major muscle flap. Red loop = Carotid artery, Blue loop = Brachiocephalic vein,* = Trachea, # = right lung apex, Arrow = Tracheoesophageal fistula, ★- Pectoralis major flap on the right side.

One patient, who received concurrent EBRT and systemic therapy with lenvatinib and pembrolizumab as part of the neoadjuvant treatment, developed a clinically significant tracheoesophageal-cutaneous fistula. This patient required a complex hemiclam-shell operation, including thyroidectomy, tumor debulking, reconstruction of the tracheoesophageal fistula with suturing of the esophagus, muscle flap placement and a tracheotomy (patient 8, **Figure 4**). Of note, intraoperatively, no residual tumor could be detected.

Due to worsening local symptoms, including dyspnea, one patient received lenvatinib 20 mg/day for only five days (patient 11). This was followed by two surgical procedures: first, a left hemithyroidectomy with tumor debulking and nine days later, a right hemithyroidectomy with tumor debulking, tracheotomy, and thrombectomy of the superior vena cava due to tumor thrombi causing upper venous congestion.

Overall, in terms of resection margins, two patients achieved an R0 resection, six patients achieved an R1 resection, and one patient had an R2 resection.

Despite demonstrating an adequate response on [18F]-FDG PET/CT, one patient declined subsequent surgical resection and opted to continue systemic therapy with lenvatinib and pembrolizumab, which had been well tolerated (patient 10).

### Adverse events during neoadjuvant treatment

In the cohort we evaluated, two patients developed tracheoesophageal and tracheocutaneous fistulas during the course of preoperative neoadjuvant therapy (patient 6 and 8). Tumor-related bleeding was not observed. Patient 6 had been treated with lenvatinib for 56 days, while patient 8 underwent simultaneous EBRT during lenvatinib treatment at an external hospital.

Other side effects associated with lenvatinib and/or pembrolizumab, dabrafenib/trametinib therapy, such as fatigue, anorexia, diarrhea, hypertension, oral mucositis, hand-foot syndrome, proteinuria, abdominal pain, hemorrhages, or pericardial effusion, were not documented during the neoadjuvant treatment. However, autoimmune hepatitis was observed in one patient three days after receiving pembrolizumab 200 mg, which required corticosteroid treatment (patient 11).

### Postoperative complications

2 of 12 patients experienced clinically relevant postoperative complications CD≥3.

One patient (patient 8), who had a clinically significant tracheoesophageal fistula preoperatively, experienced extensive wound healing problems in the previously irradiated cervical region and mediastinum. Additionally, the postoperative course was complicated by prolonged weaning over several months due to recurrent (aspiration) pneumonias.

In the second patient (patient 11), cervical re-exploration with hematoma evacuation in the previously operated left thyroid bed was performed on the first postoperative day. Due to worsening dyspnea and rapid tumor progression despite initiated systemic therapy, a completion thyroidectomy and tracheostomy were required nine days after the primary surgery. The patient ultimately died away four weeks after the initial operation (CD 5).

### Follow-up

The median follow-up period was eight months (range: 1–36 months). The median time to the first follow-up after surgery was three months (range: 1–5 months). In three patients, regular follow-up was not performed, as they either passed away during neoadjuvant therapy (patient 11) or, following neoadjuvant therapy, treatment was transitioned to best supportive care due to rapid tumor progression and a deteriorated general condition (patient 3 and 9).

At the first follow-up, all patients except one (patient 12) exhibited progressive disease, although eight out of nine patients continued systemic therapy with lenvatinib/pembrolizumab or dabrafenib/trametinib after surgery. Consequently, the systemic molecular oncology treatment for *BRAFV600E* mutated ATCs previously treated with dabrafenib/trametinib was either switched to pembrolizumab/lenvatinib, changed to radio- and/or chemotherapy, or palliative debulking surgery was performed. Two patients required tracheostomy during the course of the disease (patient 5 and 6).

**Figure 5:**
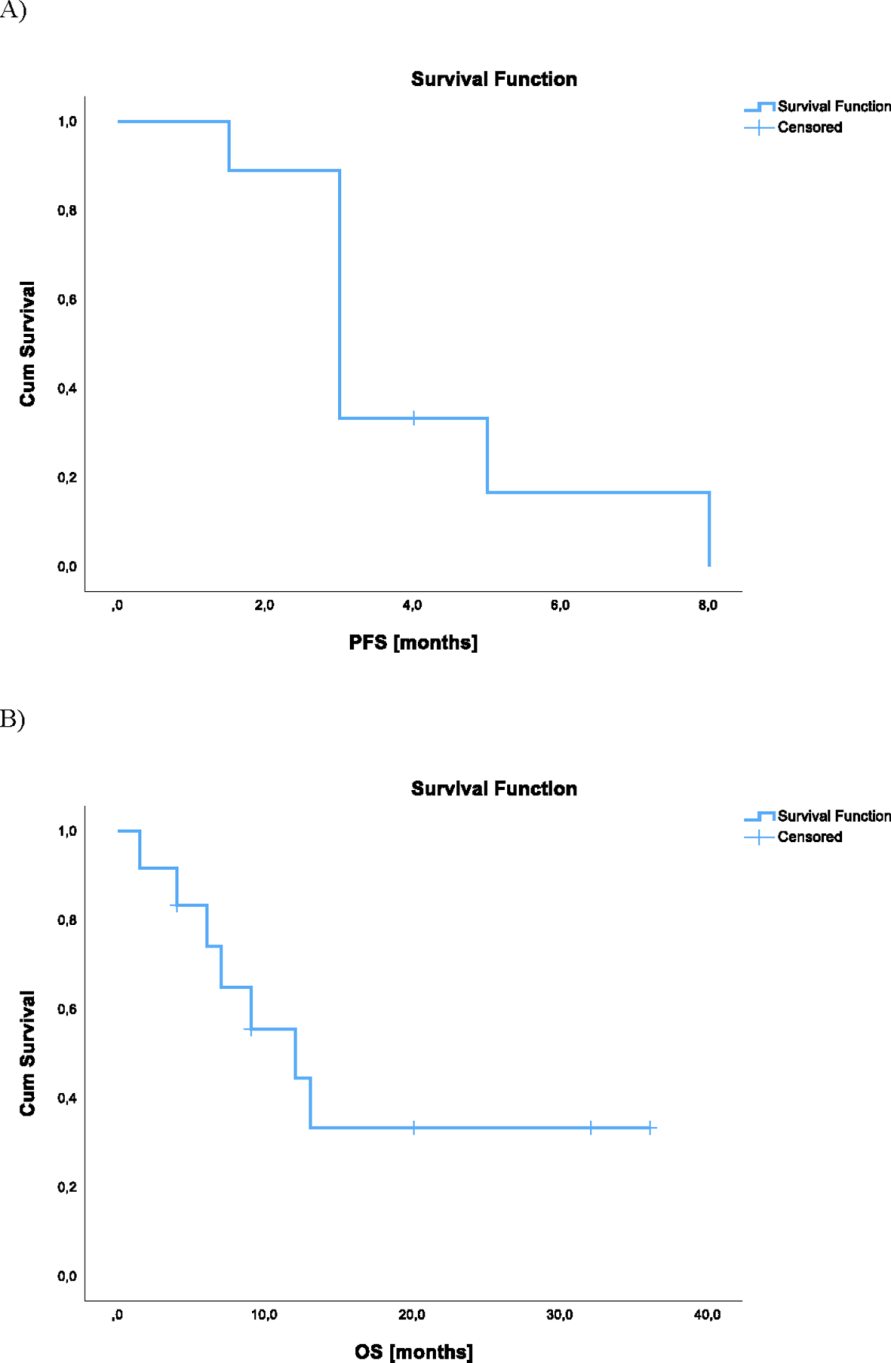
Kaplan-Meier survival curves for ATC patients receiving neoadjuvant therapy, including all three treatment regimens (dabrafenib + trametinib, lenvatinib monotherapy, and lenvatinib + pembrolizumab). **(A)** Progression free survival (PFS) analysis **(B)** Overall survival (OS) analysis.

Overall, the median PFS was three months (range: 1– not reached), and the median OS was nine months (range: 1 – not reached) (**Figure 5**). **Figure 5** presents pooled outcomes from all three neoadjuvant treatment regimens (dabrafenib plus trametinib, lenvatinib monotherapy, and lenvatinib plus pembrolizumab). While a stratified analysis by treatment group would be informative, the small sample size (n = 12) precluded meaningful subgroup comparisons. Larger prospective studies are needed to more robustly evaluate and compare the efficacy of these neoadjuvant approaches.

## Discussion

The results of this study provide further evidence supporting the feasibility of mutation-based neoadjuvant therapy in patients with initially unresectable ATC. Despite the historically poor prognosis of ATC, our findings suggest that targeted therapies can induce tumor regression, potentially enabling surgical resection and improving patient outcomes.

One of the key observations in our cohort was the conversion of initially unresectable ATC into resectable disease following neoadjuvant treatment. Previous studies have demonstrated that BRAF/MEK inhibition with dabrafenib and trametinib in *BRAFV600E* mutated ATC can result in substantial tumor shrinkage, thereby increasing the likelihood of achieving complete (R0/R1) resections (14, 15).

Wang et al. reported a case series involving six patients with initially unresectable, *BRAFV600E*-mutant anaplastic thyroid carcinoma (ATC) who were treated with neoadjuvant dabrafenib in combination with trametinib. Following targeted therapy, all patients underwent surgical resection, achieving R0 or R1 margins. Histopathological analysis of the resected specimens demonstrated a substantial reduction in viable ATC cells, and in some cases, only residual differentiated thyroid carcinoma components were identified. At six months, all patients were alive; at 12 months, the overall survival rate was 83%, with 100% local disease control (14). Similarly, the ROAR basket trial, a multicenter, non-randomized, open-label phase II study conducted by Subbiah et al., investigated the efficacy of dabrafenib plus trametinib in 36 patients with locally advanced or metastatic *BRAFV600E*-mutant ATC. The study reported an objective response rate of 56%, including three complete responses. The median PFS was 6.7 months, and the median OS was 14.5 months, representing a substantial improvement in comparison to historical outcomes in this patient population (17).

Similarly, the combination of pembrolizumab and lenvatinib has shown promising efficacy in *BRAFV600E*-wildtype ATC by modulating tumor microenvironment interactions and enhancing immune response (12, 13). Zhao et al. retrospectively analyzed 57 patients with stage IVB and IVC *BRAFV600E*-mutated ATC who received neoadjuvant *BRAFV600E* inhibitor therapy. This treatment led to tumor regression in all cases, enabling R0/R1 resections with reduced surgical extent and morbidity (15). Similarly, McCrary et al. reported tumor regression in four patients receiving neoadjuvant therapy—*BRAFV600E*-mutated tumors with dabrafenib and trametinib, and wildtype tumors with pembrolizumab and lenvatinib—allowing complete resection of a previously inoperable tumor (13). Long-term data on neoadjuvant therapy for ATC remain limited. However, Zhao et al. found the best outcomes in patients who underwent neoadjuvant *BRAFV600E*-directed therapy followed by surgery, with 12-months OS and PFS rates of 93.6% and 84.4%, respectively. Patients who had surgery first, followed by *BRAFV600E*-targeted therapy, had lower OS (74.1%) and PFS (50%), while those who did not undergo surgery had the poorest prognosis (OS 38.5%, PFS 15.4%) (15). Huang etal. conducted a systematic review of neoadjuvant targeted therapies in locally advanced thyroid cancers, which included eight ATC cases. Among these ATC patients, the overall response rate (ORR) was 87.5%, with a mean tumor size reduction of 36.5% and up to 45.5% in responders. The mean treatment-to-surgery interval was approximately 17 days, illustrating that surgical intervention remains feasible after neoadjuvant treatment (18).

Our group independently confirmed the potential of neoadjuvant therapy for advanced ATC. In the pilot proof-of-concept study, three patients with stage IVB/IVC ATC underwent successful surgery after neoadjuvant treatment (12). In the here presented extended analysis, nine out of twelve patients could undergo surgical intervention, achieving R0 or R1 resections in most cases. These findings align with emerging evidence suggesting that mutation-based neoadjuvant therapy plays a pivotal role in tumor control and may extend survival in selected patients, who also tend to develop fewer complications related to tumor infiltration of the larynx, trachea, and esophagus.

Nevertheless, our study also highlights several challenges associated with neoadjuvant treatment in ATC. While systemic therapy demonstrated efficacy in tumor shrinkage, treatment-related complications, including tracheoesophageal fistulas, were observed in two patients. This is consistent with prior reports indicating that lenvatinib, particularly in combination with EBRT, may increase the risk of fistula formation due to rapid tumor necrosis and tissue fragility (19–23). These findings underscore the importance of careful patient selection and close monitoring during treatment, particularly for individuals with extensive tumor infiltration of critical structures. The novel aspect of the therapy approach used at our institution, compared to previously reported cases and series, lies in the short duration of neoadjuvant treatment, limited to four to six weeks. This strategy aims to reduce the risk of tumor bleeding and fistula formation involving the trachea or esophagus, complications associated with prolonged lenvatinib administration.

Another notable observation in our study was the variable response to neoadjuvant therapy. While most patients demonstrated a favorable response, three exhibited disease progression or mixed responses. In one case, this may have been attributable to reduced dosing of lenvatinib due to advanced age, highlighting the need for individualized treatment regimens. Additionally, two patients did not receive pembrolizumab as initially planned due to delayed insurance approval, which resulted in treatment postponement. These administrative delays highlight a critical, often underappreciated barrier to timely and effective cancer therapy. In the context of ATC, which is characterized by rapid progression and limited treatment windows, any delay in initiating neoadjuvant therapy can lead to loss of resectability, further metastatic spread, or clinical deterioration that precludes definitive surgical intervention. The need for insurance approval, particularly for off-label or high-cost targeted therapies and immunotherapies, introduces variability into treatment timelines that may significantly influence patient outcomes. In our cohort, such delays likely contributed to suboptimal responses and poorer prognoses in the affected individuals. These findings underscore the importance of establishing fast-track approval mechanisms for life-threatening malignancies like ATC and integrating molecular diagnostics and treatment planning into a streamlined, multidisciplinary workflow. Addressing these systemic challenges is essential to ensure that the potential benefits of precision oncology can be fully realized in real-world clinical practice.

Despite these challenges, the median OS in our cohort was nine months, which is notably longer than the historically reported OS of 3–5 months for patients with unresectable ATC (3). While these results may initially appear modest, it is important to highlight that three patients (two stage IVB and one stage IVC) have already significantly exceeded the median OS, with survival durations of 20, 32 and 36 months, respectively. Two of these patients were *BRAFV600E*-positive. All three experienced early postoperative tumor progression, necessitating adjustments to their adjuvant therapies. One patient (patient 2) received chemotherapy with carboplatin and paclitaxel, achieving a complete response after six cycles. The other two patients (patient 5 and 6, both *BRAFV600E*-positive), who were treated in a palliative setting due to tumor progression after surgery, received targeted therapy with lenvatinib/pembrolizumab after progression on dabrafenib/trametinib. Additionally, debulking surgeries, tracheotomies, chemotherapy (carboplatin/paclitaxel), and/or radiotherapy were performed in their cases. These cases underscore the necessity of highly individualized and optimized adjuvant and palliative treatment strategies to improve long-term outcomes. Notably, younger patients with a *BRAFV600E* mutation appear to derive particular benefit from our therapeutic approach. Given the complexity of ATC management, patients should be treated at specialized centers to ensure access to a comprehensive range of therapeutic options. The optimal duration and combination of systemic therapies in adjuvant/palliative setting remain to be determined and warrant further investigation in prospective trials.

Neoadjuvant personalized therapy options for ATC have not been approved in Germany to date. As a result, for each patient, individualized treatment approaches had to be requested through off-label use. These applications for cost coverage often took several weeks and involved significant administrative effort. At the University Hospital Marburg, most patients were treated without waiting for the approval of cost coverage. Had the approvals been awaited, it is likely that the majority of patients would have experienced an uncontrollable tumor progression during this time. This would have undermined the concept of neoadjuvant pre-treatment planning.

Our study has several limitations, including its retrospective design and small sample size. Additionally, while our findings suggest a potential benefit of neoadjuvant targeted therapies, the lack of a control group limits direct comparisons with standard treatment approaches. Future multicenter studies with larger patient cohorts are needed to validate these findings and refine treatment algorithms for ATC.

In our cohort, residual viable ATC cells were detected in eight of nine patients who underwent surgery after neoadjuvant therapy. Only one patient (patient 8), who had received external beam radiotherapy at an external institution prior to surgery, showed no histologically detectable tumor cells in the resected specimen. Previous studies, particularly in the context of neoadjuvant *BRAFV600E-/MEK*-targeted therapy, have reported that the absence of residual ATC cells is associated with improved prognosis and longer overall survival (14). However, due to the small number of complete pathological responses in our study, no outcome-based conclusions can be drawn. Future prospective investigations with standardized histopathological assessment and molecular correlation are warranted to better define the prognostic significance of histopathological residual tumor burden after neoadjuvant treatment.

In conclusion, this study supports the role of mutation-based neoadjuvant therapy as a promising approach in the management of initially unresectable ATC. While challenges such as treatment-related complications and variable responses exist, our findings highlight the potential for improved resectability and prolonged survival in selected patients. Further research is required to optimize treatment strategies and identify predictive markers of response to enhance patient selection.

## Data Availability

The original contributions presented in the study are included in the article/**Supplementary Material**. Further inquiries can be directed to the corresponding author.

## References

[1] Bible KC Kebebew E Brierley J Brito JP Cabanillas ME Clark TJ Jr. 2021 american thyroid association guidelines for management of patients with anaplastic thyroid cancer. Thyroid: Off J Am Thyroid Assoc. (2021) 31:337–86. doi: 10.1089/thy.2020.0944, PMID: 33728999 PMC8349723

[2] Haddad RI Lydiatt WM Ball DW Busaidy NL Byrd D Callender G. Anaplastic thyroid carcinoma, version 2.2015: clinical practice guidelines in oncology HHS public access. J Natl Compr Canc Netw. (2015) 13:1140–50. doi: 10.6004/jnccn.2015.0139, PMID: 26358798 PMC4986600

[3] Wächter S Vorländer C Schabram J Mintziras I Fülber I Manoharan J. Anaplastic thyroid carcinoma: changing trends of treatment strategies and associated overall survival. Eur Arch Oto-rhino-laryngology: Off J Eur Fed Oto-Rhino-Laryngol Societies (EUFOS): Affiliated German Soc Oto-Rhino-Laryngol Head Neck Surg. (2020) 277:1507–14. doi: 10.1007/s00405-020-05853-8, PMID: 32060602

[4] Dralle H Musholt TJ Schabram J Steinmüller T Frilling A Simon D. German Association of Endocrine Surgeons practice guideline for the surgical management of Malignant thyroid tumors. Langenbecks Arch Surg. (2013) 398:347–75. doi: 10.1007/s00423-013-1057-6, PMID: 23456424

[5] Goffredo P Thomas SM Adam MA Sosa JA Roman SA. Impact of timeliness of resection and thyroidectomy margin status on survival for patients with anaplastic thyroid cancer: an analysis of 335 cases. Ann Surg Oncol. (2015) 22:4166–74. doi: 10.1245/s10434-015-4742-6, PMID: 26271394

[6] Kebebew E Greenspan FS Clark OH Woeber KA McMillan A. Anaplastic thyroid carcinoma. Treatment outcome and prognostic factors. Cancer. (2005) 103:1330–5. doi: 10.1002/cncr.20936, PMID: 15739211

[7] Lin B Ma H Ma M Zhang Z Sun Z Hsieh IY. The incidence and survival analysis for anaplastic thyroid cancer: a SEER database analysis. Am J Trans Res. (2019) 11:5888–96., PMID: 31632557 PMC6789224

[8] Maniakas A Dadu R Busaidy NL Wang JR Ferrarotto R Lu C. Evaluation of overall survival in patients with anaplastic thyroid carcinoma, 2000-2019. JAMA Oncol. (2020) 6:1397. doi: 10.1001/jamaoncol.2020.3362, PMID: 32761153 PMC7411939

[9] Higashiyama T Sugino K Hara H Ito KI Nakashima N Onoda N. Phase II study of the efficacy and safety of lenvatinib for anaplastic thyroid cancer (HOPE). Eur J Cancer. (2022) :173:210–218. doi: 10.1016/j.ejca.2022.06.044, PMID: 35932627

[10] Dierks C Seufert J Aumann K Ruf J Klein C Kiefer S. Combination of lenvatinib and pembrolizumab is an effective treatment option for anaplastic and poorly differentiated thyroid carcinoma. Thyroid. (2021) 31:1076–85. doi: 10.1089/thy.2020.0322, PMID: 33509020 PMC8290324

[11] Cabanillas ME Dadu R Ferrarotto R Liu S Fellman BM Gross ND. Atezolizumab combinations with targeted therapy for anaplastic thyroid carcinoma (ATC). J Clin Oncol. (2020) 38:6514. doi: 10.1200/JCO.2020.38.15_suppl.6514

[12] Maurer E Eilsberger F Wächter S Knorrenschild JR Pehl A Holzer K. Mutation-based, short-term “neoadjuvant” treatment allows resectability in stage IVB and C anaplastic thyroid cancer. Eur Arch oto-rhino-laryngology: Off J Eur Fed Oto-Rhino-Laryngol Societies (EUFOS): Affiliated German Soc Oto-Rhino-Laryngol Head Neck Surg. (2023) 280:1509–18. doi: 10.1007/S00405-023-07827-Y, PMID: 36637521 PMC9899736

[13] McCrary HC Aoki J Huang Y Chadwick B Kerrigan K Witt B. Mutation based approaches to the treatment of anaplastic thyroid cancer. Clin Endocrinol. (2022) 96:734–42. doi: 10.1111/cen.14679, PMID: 35067961

[14] Wang JR Zafereo ME Dadu R Ferrarotto R Busaidy NL Lu C. Complete surgical resection following neoadjuvant dabrafenib plus trametinib in BRAF ^V600E^ -mutated anaplastic thyroid carcinoma. Thyroid. (2019) 29:1036–43. doi: 10.1089/thy.2019.0133, PMID: 31319771 PMC6707029

[15] Zhao X Wang JR Dadu R Busaidy NL Xu L Learned KO. Surgery after BRAF-directed therapy is associated with improved survival in BRAF^V600E^ mutant anaplastic thyroid cancer: A single-center retrospective cohort study. Thyroid. (2023) 33:484–91. doi: 10.1089/thy.2022.05.04, PMID: 36762947 PMC10122263

[16] Clavien PA Barkun J de Oliveira ML Vauthey JN Dindo D Schulick RD. The Clavien-Dindo classification of surgical complications: five-year experience. Ann Surg. (2009) 250:187–96. doi: 10.1097/SLA.0b013e3181b13ca2, PMID: 19638912

[17] Subbiah V Kreitman RJ Wainberg ZA Cho JY Schellens JHM Soria JC. Dabrafenib plus trametinib in patients with BRAF V600E-mutant anaplastic thyroid cancer: updated analysis from the phase II ROAR basket study. Ann Oncol. (2022) 33:406–15. doi: 10.1016/j.annonc.2021.12.014, PMID: 35026411 PMC9338780

[18] Huang NS Wang Y Wei WJ Wang Y Wei WJ Xiang J Chen JY Guan Y. A systematic review of neoadjuvant targeted therapy in locally advanced thyroid cancer. Holist Integ Oncol. (2022) 1:16. doi: 10.1007/s44178-022-00016-7

[19] Obata K Sugitani I Ebina A Sugiura Y Toda K Takahashi S. Common carotid artery rupture during treatment with lenvatinib for anaplastic thyroid cancer. Int Cancer Conf J. (2016) 5:197–201. doi: 10.1007/s13691-016-0257-7, PMID: 31149454 PMC6498322

[20] Staub Y Nishiyama A Suga Y Fujita M Matsushita R Yano S. Clinical characteristics associated with lenvatinib-induced fistula and tumor-related bleeding in patients with thyroid cancer. Anticancer Res. (2019) 39:3871–8. doi: 10.21873/anticanres.13537, PMID: 31262915

[21] Suyama K Murakami D Fujiwara S Takeshita T Sueta A Toukolnao M. Massive arterial bleeding after lenvatinib therapy for thyroid cancer. Int J Cancer Clin Res. (2016) 3:074. doi: 10.23937/2378-3419/3/6/1074

[22] Blevins DP Dadu R Hu M Baik C Balachandran D Ross W. Aerodigestive fistula formation as a rare side effect of antiangiogenic tyrosine kinase inhibitor therapy for thyroid cancer. Thyroid. (2014) 24:918–22. doi: 10.1089/thy.2012.0598, PMID: 24635127 PMC4026371

[23] Valerio L Giani C Agate L Molinaro E Viola D Bottici V. Prevalence and risk factors of developing fistula or organ perforation in patients treated with lenvatinib for radioiodine-refractory thyroid cancer. Eur Thyroid J. (2021) 10:399–407. doi: 10.1159/000514182, PMID: 34540710 PMC8406256

