# Supplementary material for: Mutation-based, neoadjuvant treatment for advanced anaplastic thyroid carcinoma
Source: Front Endocrinol (Lausanne). 2025 Aug 25;16:1619875. doi: 10.3389/fendo.2025.1619875 (PMC12414790; doi:10.3389/fendo.2025.1619875)
Supplement: Supplementary file 1 [file Table1.docx]

| **Pat.**  **Supplementary material**  Table 1. *BRAFV600E*-mutation status, selected NGS results, and clinical course during neoadjuvant therapy  Table 1. *BRAFV600E*-mutation status, selected NGS results, and clinical course during neoadjuvant therapy | ***BRAFV600E*-Mutation**  **Status** | **Results of NGS** | **Change in Tumor Size (Pre-Post neoadjuvant therapy) *** | **Time from initiation of neoadjuvant therapy to surgery (days)** |
| --- | --- | --- | --- | --- |
| 1 | Negative | NTRK/RET/KRAS/NRAS negative | - 12 % | 28 |
| 2 | Negative | NRAS mutation detected; KRAS/ALK/ROS1/RET/  MET/NTRK negative | - 32 % | 28 |
| 3 | Negative | CREB3L2-PPARG fusion detected; TP53/CDKN2A/RET/HRAS/PIK3CA/PTEN/KRAS/ATM/TERT/NTRK/ROS1/ALK/  MEK negative | - 7 % * | No surgery |
| 4 | Negative | TP53 mutation detected; CDKN2A/RET/HRAS/  PIK3CA/PTEN/KRAS/ATM/TERT/NTRK/ROS1/ALK/  MEK negative | - 20 % | 42 |
| 5 | Positive | NRAS/ALK/RET/NTRK/ROS1/  MET negative | - 29 % | 35 |
| 6 | Positive | **TP53/CDKN2A/RET/HRAS/ PIK3CA/PTEN/KRAS/ATM/ TERT/NTRK/ROS1/ALK/MEK negative** | - 32 % | 56 |
| 7 | Negative | TP53/PTEN/ATM mutations detected; CDKN2A/RET/HRAS/  PIK3CA/KRAS/TERT/ALK/  NTRK negative | + 13 % | 35 |
| 8 | Negative | NTRK/RET/KRAS/NRAS/APC/  TP53 negative | - 43 % | 31 |
| 9 | Negative | NRAS/TP53 mutations detected; RET negative | - 17 % * | No surgery |
| 10 | Negative | TP53/NRAS/KMT2C mutations detected; CDKN2A/RET/HRAS/  PIK3CA/PTEN/KRAS/ATM/  TERT negative | - 27 % | No surgery |
| 11 | Negative | Not performed | Not performed | 5 |
| 12 | Negative | ATM/NF2/KMT2C mutations detected; TFG/ADGRG7 fusions detected; TP53/CDKN2A/RET/HRAS/  PIK3CA/PTEN/KRAS/TERT/  NTRK/ROS1/ALK/MEK negative | - 1 % | 28 |

Pat. = Patient, NGS = next-generation sequencing

* In these patients, a partial reduction in tumor volume was observed. However, this morphological regression was minor and did not correspond to a decrease in metabolic activity as assessed by [18F]-FDG PET/CT. Therefore, despite the slight size reduction, no adequate response to neoadjuvant therapy can be assumed. It is important to note that minor morphological changes may underestimate the true metabolic response in [18F]-FDG PET/CT imaging. All measurements were performed according to standardized protocols.
